# High GPR56 surface expression correlates with a leukemic stem cell gene signature in CD34‐positive AML

**DOI:** 10.1002/cam4.2053

**Published:** 2019-03-07

**Authors:** Shruti Daga, Angelika Rosenberger, Franz Quehenberger, Nina Krisper, Barbara Prietl, Andreas Reinisch, Armin Zebisch, Heinz Sill, Albert Wölfler

**Affiliations:** ^1^ Division of Hematology Medical University of Graz Graz Austria; ^2^ CBmed Center of Biomarker Research in Medicine Graz Austria; ^3^ Institute of Medical Informatics, Statistics and Documentation Medical University of Graz Graz Austria; ^4^ Division of Endocrinology and Diabetology Medical University of Graz Graz Austria

**Keywords:** acute myeloid leukemia, CLL‐1, gene expression signature, GPR56, leukemic stem cells

## Abstract

Acute myeloid leukemia (AML) is driven by a minor fraction of leukemic stem cells (LSCs) whose persistence is considered being the primary cause of disease relapse. A detailed characterization of the surface immunophenotype of LSCs to discriminate them from bulk leukemic blasts may enable successful targeting of this population thereby improving patient outcomes in AML. To identify surface markers, which may reflect LSC activity at diagnosis, we performed a detailed analysis of 16 putative LSC markers in CD34/38 leukemic subcompartments of 150 diagnostic AML samples using multicolor flow cytometry. The most promising markers were then selected to determine a possible correlation of their expression with a recently published LSC gene signature. We found GPR56 and CLL‐1 to be the most prominently differently expressed surface markers in AML subcompartments. While GPR56 was highest expressed within the LSC‐enriched CD34^+^38^−^ subcompartment as compared to CD34^+^38^+^ and CD34^−^ leukemic bulk cells, CLL‐1 expression was lowest in CD34^+^38^−^ leukemic cells and increased in CD34^+^38^+^ and CD34^−^ blasts. Furthermore, high GPR56 surface expression in CD34^+^38^−^ leukemic cells correlated with a recently published LSC gene expression signature and was associated with decreased overall survival in patients receiving intensive chemotherapy. In contrast, CLL‐1 expression correlated inversely with the LSC gene signature and was not informative on outcome. Our data strongly support GPR56 as a promising clinically relevant marker for identifying leukemic cells with LSC activity at diagnosis in CD34‐positive AML.

## 1 INTRODUCTION

Acute myeloid leukemia (AML) is characterized by a hierarchical cellular organization, with a minor fraction of self‐renewing and probably chemotherapy‐resistant leukemic stem cells (LSCs) at the apex of this hierarchy.^1^, ^2^, ^3^ Within CD34‐positive leukemias, which comprise about three quarters of all AMLs, LSCs have been shown to predominantly reside in the CD34^+^38^−^ cell fraction.^4^, ^5^ Since LSCs are considered being the primary cause of disease relapse in AML, successful targeting of this population is crucial to improve patient outcomes. Given this importance, a detailed characterization of the surface immunophenotype of LSCs to discriminate them from bulk leukemic blasts has been of great interest. Several surface markers including CD47, CD117, CD123, CLL‐1, TIM‐3, IL1RAP, and JAM‐C have been reported to be up‐regulated on CD34^+^38^−^ LSCs or to mark AML cells with high repopulating activity in immunocompromised mice.^3^, ^6^, ^7^, ^8^ Recently, GPR56 (G‐protein coupled receptor 56) and CLL‐1 (C‐type lectin‐like molecule 1, also known as CLEC12A) have drawn particular attention. While expression of GPR56 allowed identification of AML cells with high repopulating potential irrespective of CD34 and CD38 expression levels,^9^ CLL‐1 has emerged as an attractive target for antibody‐ or chimeric antigen receptor (CAR)‐based therapeutics, since it is hardly expressed on normal hematopoietic stem and progenitor cells (HSPCs)^10^: A bispecific anti‐CD3/anti‐CLL‐1 antibody was shown to selectively kill CLL‐1‐expressing AML cell lines in in vitro co‐cultures as well as CLL‐1‐expressing monocytes in humanized mice and monkeys.^11^ Comparably, a recently developed anti‐CLL‐1 pyrrolobenzodiazepine antibody‐drug conjugate exhibited cytotoxicity in xenograft mouse models using CLL‐1‐expressing AML cell lines and CLL‐1 expressing monocytes and neutrophils in cynomolgus monkeys, but lacked target independent toxicities.^12^ In addition, two groups reported the generation of CLL‐1‐specific CAR T cells, which efficiently lysed CLL‐1 expressing AML cells in vitro as well as in mice xenografted with the HL60 or U937 AML cell lines.^13^, ^14^

To identify surface markers, which may reflect LSC activity at diagnosis, we performed a detailed analysis of sixteen putative LSC markers in CD34/38 leukemic subcompartments using multicolor flow cytometry. The most promising markers were then selected to determine a possible correlation of their expression with a recently published LSC gene signature.^15^ Finally, expression levels of these surface markers were analyzed for their impact on survival in AML patients receiving intensive chemotherapy.

## 2 MATERIALS AND METHODS

### 2.1 Clinical samples

A total of 150 adults diagnosed with AML according to WHO criteria at the Division of Hematology, Medical University of Graz, Austria were included in this study. Patient characteristics are shown in Supporting information, Table S1. Bone marrow (BM) or peripheral blood (PB) samples with blast counts >20% were collected from patients at diagnosis and processed as described previously.^16^, ^17^ In 25 cases, samples were also available at relapse. To assess the normal CD34^+^ HSPC compartments, normal bone marrow samples (NBM) were obtained from 16 lymphoma patients without any evidence of disease in the bone marrow. Information on clinical data such as white blood cell counts, cytogenetic risk stratification, treatment as well as outcome parameter were collected from medical records and the electronic documentation program MEDOCS (Medical Documentation and Communication System, SAP Germany). Informed consent was obtained from all patients, and the study has been approved by the Institutional Review Board of Medical University Graz, Austria (protocol 26‐050 ex 13/14 and 29‐499 ex 16/17).

### 2.2 Flow cytometry analysis and sorting

Twelve‐color multiparameter flow cytometry was performed using a 4‐laser Fortessa cytometer (Becton Dickinson; BD; San Jose, CA) with strictly harmonized baseline settings. At time of analysis, cryopreserved cells were thawed, washed with phosphate‐buffered saline, and stained with the appropriate antibodies (see Supporting information, Table S2). At least 200 000 events were recorded, and data were analyzed using either Kaluza software (Beckman Coulter, Brea, CA, USA) or for merging all panels using the Infinicyt software (Cytognos, Salamanca, Spain). CD34, CD38, and CD45 served as backbone markers in all panels. Appropriate isotype controls were used to determine the level of background staining.

Leukemic cells were identified based on low expression of CD45 and low side scatter. The cellular compartments of CD34‐positive AMLs were defined by expression of CD34/CD38, and the expression of tested markers was analyzed on these individual cellular compartments (for gating see Supporting information, Figure S1). The percentage of marker‐positive cells and the mean fluorescence intensities (MFI) for each population were recorded.

Cell sorting was performed under sterile conditions using a Becton Dickinson Aria II cell sorter. The CD34^+^38^−^ leukemic cells of twelve CD34‐positive AML samples were sorted based on expression of GPR56 and CLL1 into four fractions as follows: CD34^+^38^‐^GPR56^hi^; CD34^+^38^−^GPR56^lo^; CD34^+^38^−^CLL‐1^hi^, and CD34^+^38^−^CLL‐1^lo^. Figure S2 in supporting information shows the sorting strategy.

### 2.3 Expression analysis of LSC17 genes^15^

RNA was extracted from sorted cells using the RNeasy Micro Kit (Qiagen, Hilden, Germany), and cDNA was synthesized from 35ng RNA using the Reverse transcription kit (Applied Biosystems, Foster City, CA) according to manufacturer's instructions. Quantitative PCR was performed using the ABI Prism 7700 Sequence Detector (Applied Biosystems). All PCR reactions were performed in triplicates using TaqMan Gene Expression mastermix (Applied Biosystems). For analysis, the genes *GAPDH* and *RPL13A* were used as internal control. The following Taqman Probes were purchased from Applied Biosystems: *GAPDH* (Hs04194366_g1); *RPL13A* (Hs02786624_g1); *CD34* (Hs02576480_m1); *GPR56* (Hs00938474_m1); *ZBTB46* (Hs01008166_m1); *MMRN1* (Hs01113299_m1); *CPXM1* (Hs00219709_m1); *DPYSLR3* (Hs00181668_m1); *SMIM24* (Hs00415400_m1); *BEX3* (Hs00276273_s1); *DNMT3B* (Hs00171876_m1); *CDK6* (Hs01026371_m1); *SOCS2* (Hs00919620_m1); *AKR1C3* (Hs00366267_m1); *ARHGAP22* (Hs01098342_m1); *LAPTM4B* (Hs00363282_m1); *EMP1* (Hs00608055_m1); *KIAA0125* (Hs00796164_s1); *NYNRIN* (Hs00394058_m1). The ΔCt values were obtained for every probe after normalization from internal control. A ratio of expression levels of genes in GPR56^hi^ vs GPR56^lo^ cells as well as CLL‐1^hi^ vs CLL‐1^lo ^cells was calculated.

### 2.4 Statistical analysis

Differences in characteristics of patients were calculated using a two‐sided Fisher´s exact or Mann‐Whitney test. Comparison between two groups concerning MFI values, percentages of marker‐positive cells, and gene expression values was done using the Mann‐Whitney test in unpaired samples and using the Wilcoxon signed‐rank test in paired samples. Comparison between more than two groups in paired samples was done using the Kruskal‐Wallis test. The Kaplan‐Meier method was applied to generate the survival curves, and differences were assessed by the log‐rank test. All statistical analyses were carried using GraphPad Prism software version 7.0 (GraphPad Software, La Jolla, CA) and R 3.4.0 (www.r-project.org). All hypothesis testing was carried out for alpha = 0.05.

## 3 RESULTS

### 3.1 GPR56 and CLL‐1 were the most prominently differently expressed surface markers in leukemic subcompartments with highest GPR56 expression levels in the CD34^+^38^−^ LSC‐containing subpopulation

**Figure 1:**
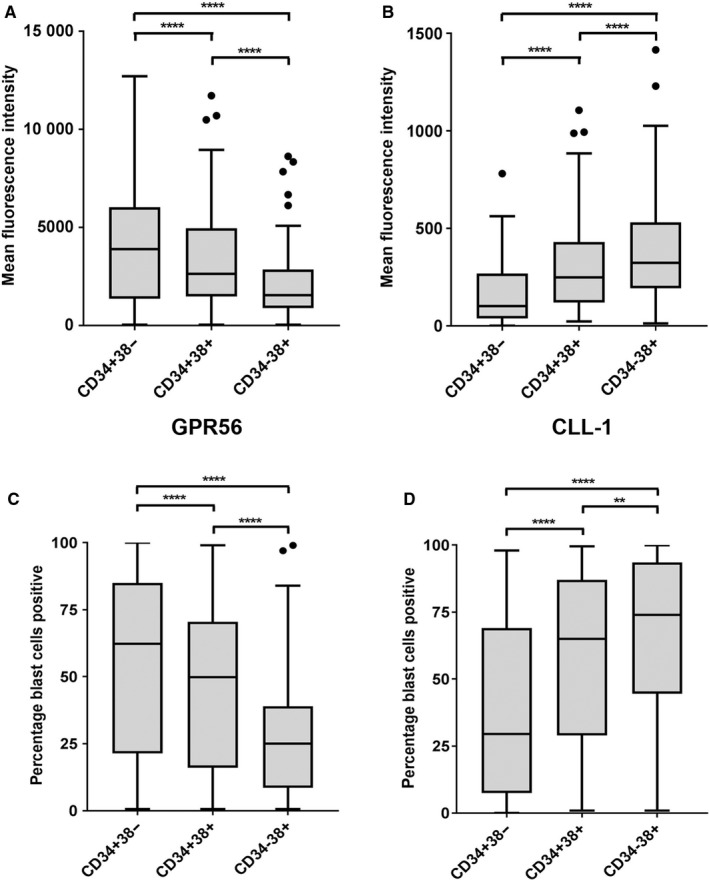
Surface expression (A and B) and percentage (C, D) of GPR56‐ and CLL‐1‐positive cells in CD34/38 compartments of AML samples. Mean fluorescence intensity (MFI) of GPR56 (A) and CLL‐1 (B) surface expression in CD34/38 compartments of AML samples at diagnosis (n = 57). GPR56 and CLL‐1 expression was highly significantly different between all groups as assessed by the Kruskal‐Wallis test (*P* < 0.0001) as well as when tested between two groups. Percentage of blasts positive for GPR56 (C) and CLL‐1 (D) surface expression in CD34/38 compartments of AML samples at diagnosis (n = 57). Percentage of blasts positive for GPR56 or CLL‐1 expression was highly significantly different between all groups as assessed by the Kruskal‐Wallis test as well as when tested between two groups by the Wilcoxon rank test (**P < 0.01; ****P < 0.0001)

Among 150 samples from adults with newly diagnosed AML (patient characteristics are shown in Supporting information, Table S1), 108 (72%) were tested CD34 positive (defined by at least 2% CD34‐expressing leukemic blasts within the bulk leukemia population to exclude major contamination of this population by normal HSPCs, which may comprise a population of up to one percent also at AML diagnosis). The most prominently differentially expressed surface markers were GPR56 and CLL‐1. While GPR56 displayed an elevated expression as calculated by mean fluorescence intensity (MFI) values in CD34‐positive AML specimens (*P* < 0.001, see Supporting information, Figure S3A), CLL‐1 expression was higher in CD34‐negative AMLs (*P* < 0.0001, Supporting Information, Figure S3B). Other markers, which were differentially expressed between CD34‐positive and ‐negative samples, are shown in Table S3. Within the CD34‐positive AMLs, 57 samples fully displayed all three compartments concerning differential CD34/38 expression (CD34^+^38^−^, CD34^+^38^+^, and CD34^‐^38^+ ^subcompartment; for gating see Supporting information, Figure S1). GPR56 MFI levels were highest in the CD34^+^38^−^ LSC‐containing subpopulation, lower in the CD34^+^38^+^ cells, and lowest in the CD34^‐^ compartment (*P* < 0.0001, Figure 1A). Accordingly, the percentage of GPR56+ cells was also highest in the CD34^+^38^‐^ subpopulation and lowest in the CD34^−^ compartment (54.9 ± 34.3% vs 45.3 ± 30.4% vs 29.0 ± 26.1%; *P* < 0.001, Figure 1C). In contrast, CLL‐1 was significantly up‐regulated in the more mature CD34^+^38^+^ and CD34^−^ leukemic cells as compared to the CD34^+^CD38^‐^ LSC‐containing compartment in CD34‐positive AMLs, as seen by MFI values (*P* < 0.0001, Figure 1B) as well as percentage of CLL‐1^+^ cells (38.4 ± 33.2 vs 58.7 ± 31.5 vs 65.4 ± 31.2%; *P* < 0.001, Figure 1D). Among other markers tested, CD99 (*P* < 0.01), CD117 (*P* < 0.01), CD123 (*P* < 0.05), and CD44 (*P* < 0.05) were also differentially expressed among CD34/38 leukemic subpopulations (Supporting information, Table S4).

Next, we compared GPR56 and CLL‐1 surface expression levels between CD34^+^38^‐^ leukemic cells and their normal HSPC counterparts. While CLL‐1 was hardly expressed in CD34^+^38^−^ HSPCs,^10^, ^11^, ^12^ GPR56 was present on the majority of these cells with slightly, but not significantly lower MFI values as compared to CD34^+^38^−^ leukemic cells (*P* = 0.42, Supporting Information, Figure S4) as also reported previously.^9^, ^20^, ^21^ Analysis of paired diagnostic and relapse samples (n = 25) indicated that both GPR56 and CLL‐1 expression were conserved throughout the disease course in most cases (Supporting information, Figure S5).

### 3.2 High GPR56 surface expression correlates with an LSC‐associated gene expression profile and confers adverse outcome

**Figure 2:**
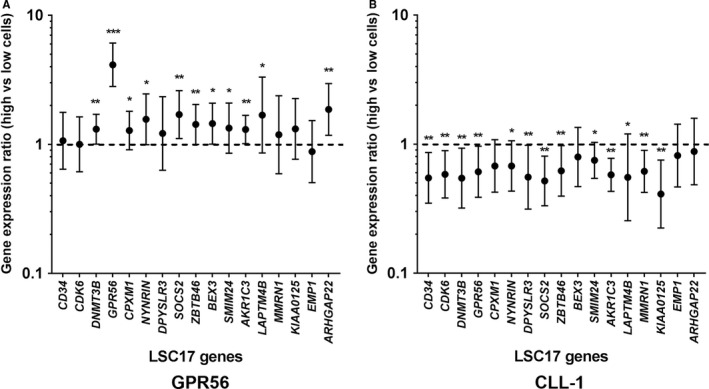
Expression ratios of LSC17 genes in CD34^+^CD38^‐^ AML cells sorted according to their GPR56 and CLL‐1 surface expression. A, Mean geometric gene expression ratios (± geometric standard deviation) of LSC17 genes in sorted GPR56^hi^CD34^+^38^−^ vs GPR56^lo^CD34^+^38^−^ AML cells (n = 12); ratios >1 indicate higher expression of the respective gene in GPR56^hi^CD34^+^38^−^ cells; *P < 0.05, **P < 0.01, ***P < 0.001 by the Wilcoxon rank test. B, Mean geometric gene expression ratios of LSC17 genes in sorted GPR56^hi^CD34^+^38^−^ vs GPR56^lo^CD34^+^38^−^ AML cells (n = 12)

In a recent comprehensive analysis of LSC gene expression signatures encompassing 78 AML patient samples, Ng et al^15^ identified genes, which were significantly up‐regulated in LSC‐containing leukemic cell fractions when compared to nonengrafting blast populations. A score based on the expression of the 17 most informative genes (LSC17 score), which interestingly also included the *GPR56 *gene, was strongly associated with poor overall survival in several AML cohorts.^15^ To test whether GPR56 or CLL‐1 surface expression correlates with this LSC17 gene signature, we sorted CD34^+^38^−^ leukemic cells according to their GPR56 as well as CLL‐1 surface levels (Supporting Information, Figure S2) and determined the expression of genes included in this LSC17 panel by quantitative RT‐PCR (qPCR). While eleven out of 17 genes were significantly up‐regulated in GPR56^hi^ vs GPR56^lo ^CD34^+^38^‐^ leukemic cells (Figure 2A), none of the genes was higher expressed in CLL‐1^hi ^as compared to CLL‐1^lo ^CD34^+^38^−^ leukemic cells (Figure 2B). In contrast, 13 out of 17 genes were significantly lower expressed in CLL‐1^hi ^as compared to CLL‐1^lo ^CD34^+^38^−^ leukemic cells. Using a global statistical analysis to test for an association of groups of genes with a phenotypical parameter,^23^ expression of these LSC genes was highly significantly associated with high GPR56 surface expression in CD34^+^38^−^ leukemic cells (*P* < 0.0001), even when the *GPR56* qPCR data were omitted (*P* < 0.001, Supporting Information, Figure S6). In contrast, CLL‐1 expression correlated inversely with this LSC gene signature in CD34^+^38^−^ AML cells.

**Figure 3:**
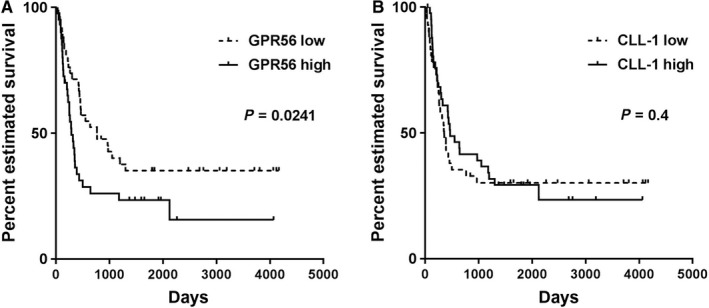
Overall survival in AML patients receiving intensive chemotherapy according to GPR56 and CLL‐1 surface expression. Overall survival according to GPR56 (A) and CLL‐1 (B) surface expression levels in AML patients receiving intensive chemotherapy (n = 84). While high GPR56 expression was associated with worse overall survival (median overall survival 284 days vs 769 days, *P* < 0.05 in the log‐rank test), CLL‐1 expression levels were not informative

Since a leukemic stemness gene expression signature has been associated with a worse outcome in AML patients,^15^, ^20^ we next analyzed overall survival in 84 patients of our cohort receiving intensive chemotherapy ± allogeneic stem cell transplantation in relation to their GPR56 and CLL‐1 surface expression status. Indeed, high GPR56 surface expression at diagnosis was associated with significantly lower OS (median OS 284 vs 769 days; *P* = 0.0241, Figure 3A), while expression of CLL‐1 did not show prognostic significance (median OS 463 vs 352 days; *P* = 0.4, Figure 3B). Interestingly, patients within the highest quartile of GPR56 expression had a lower complete remission rate after first induction chemotherapy as all other patients (38% vs 68%) suggesting that high GPR56 expression might be associated with resistance to chemotherapy. However, when tested in a multivariate analysis including cytogenetic risk, leukocyte counts, type of leukemia, and receipt of an allogeneic stem cell transplantation GPR56 expression did not remain significant (Supporting information, Table S5) probably to the rather low number of patients in our cohort as well as to its known correlation with adverse cytogenetic markers.^9^

## 4 DISCUSSION

In this comprehensive analysis of putative LSC marker, we have identified GPR56 and CLL‐1 to be the most prominently differently expressed surface markers in AML CD34/38 subcompartments at diagnosis. While GPR56 was highest expressed within the LSC‐enriched CD34^+^38^−^ subcompartment as compared to CD34^+^38^+^ and CD34^−^ leukemic bulk cells, CLL‐1 expression was lowest in CD34^+^38^−^ leukemic cells and increased in CD34^+^38^+^ and CD34^−^ blasts. In addition, high GPR56 surface expression in CD34^+^38^−^ leukemic cells correlated with a recently published LSC gene expression signature.^15^ These results clearly indicate that high GPR56 surface expression allows identification of AML cells with an LSC‐associated gene expression profile and therefore confirm and extend data from Pabst et al,^9^ who could show high GPR56 expression to be correlated with high repopulation activity of leukemic cells in immunocompromised mice irrespective of their CD34/38 status. Interestingly, in several gene expression studies aiming at identifying a “stemness signature” of leukemic cell subpopulations GPR56 expression was found to be related to LSC function. For example, expression of GPR56 was higher in the LSC‐enriched fractions in comparison to the nonengrafting bulk leukemic cells with a clear increase in CD34^+^ LSCs as compared to the CD34^−^ nonengrafting leukemic cells.^15^ A complementary study found that GPR56 expression was highest in the CD34^+^ LSC progeny with a LMPP and GMP phenotype.^22^ Thus, flow cytometry analysis of GPR56 surface expression may be helpful in determining the pool of AML cells with LSC activity at diagnosis. This is of clinical importance, since the number of LSC^24^, ^25^ as well as leukemic stemness gene expression signatures in AML cells^15^, ^26^, ^27^ were associated with a worse clinical outcome. Interestingly, GPR56 RNA expression levels also correlated with treatment outcome in two independent prospective clinical trials of the Austrian‐German Study group encompassing 423 patients.^22^ Using the median expression level of GPR56 as cutoff, high GPR56 expression was associated with lower event‐free and overall survival. In accordance, RNA‐seq data from Pabst et al revealed that the GPR56 high expressing group of AML patients had a poorer overall survival.^9^ In the present study, we found that overall survival in patients receiving intensive chemotherapy was affected by their GPR56 protein expression status at diagnosis, since patients with a high GPR56 expression as assessed by flow cytometry showed an inferior overall survival in univariate analysis. However, due to our rather small cohort more comprehensive studies are needed to establish a definite role of GPR56 surface expression as an independent adverse risk factor in AML outcome.

A role of GPR56 in AML was first described in EVI1^high^ AML, wherein EVI1 was shown to directly bind to the promoter region of GPR56.^20^ GPR56 knockdown in EVI1^high^ AML cell lines reduced viability, and the cells displayed increased susceptibility to chemotherapy drugs.^20^ In an AML cohort encompassing 179 patients, GPR56 mRNA expression levels correlated with the expression of drug efflux transporters ABCG1, ABCC1, and ABCA2 indicating an association of GPR56 expression and drug resistance.^9^ In other tissues surface GPR56 was shown to exert its cellular functions by interacting with protein ligands present in the extracellular matrix, such as collagen III and tissue transglutaminase 2.^28^, ^29^ It was therefore speculated that GPR56 is involved in adhesion and repopulating activity of HSPCs as well as LSCs^20^, ^22^ indicating a probable role for GPR56 in the crosstalk between LSCs and their microenvironmental niche mediating chemoresistance. In line with these mechanisms, we found lower CR rates after first induction chemotherapy in patients within the highest quartile of GPR56 expression.

Given these data and its surface expression in AML cells with LSC activity, GPR56 might represent an interesting target for antibody‐directed therapy. However, as reported in this study as well as others, GPR56 is also expressed on normal HSPCs^9^, ^20^, ^21^ and other tissues,^28^, ^29^ which might hamper its therapeutic targeting. Concerning the hematopoietic system GPR56 knockout mice were reported to have lower numbers of HSPCs in comparison to wild type mice.^20^ However, another group did not detect a significant effect of GPR56 deficiency on function and maintenance of HSPCs in mice.^21^ In a recent paper, Daria et al indeed demonstrated that the human AML cell line MV4‐11 as well as primary AML patient samples were efficiently targeted by a blocking anti‐GPR56 antibody resulting in a major reduction of engraftment potential in immunocompromised mice.^22^ These observations are encouraging, although future investigations will have to show whether normal human HSPC engraftment depends on GPR56 to the same extent as human LSCs.

In contrast to GPR56, CLL‐1 is hardly expressed on normal HSPCs with the exception of committed myeloid (progenitor) cells. Accordingly, CLL‐1 has not been implicated in stem cell biology and CLL‐1^+^ HSPCs do not contain colony‐forming cells in long‐term colony initiating cell assay (LTC‐IC).^31^ In contrast, in AML CLL‐1 was initially reported to be expressed in CD34^+^38^‐^ LSC in a study encompassing 89 AML samples. In three samples tested CD34^+^CLL‐1^+^ AML cells were indeed able to engraft and generate CLL‐1^+^ blasts in immunodeficient mice.^10^ Thus, CLL‐1 has emerged as an attractive target for antibody‐ or CAR T‐cell‐based therapeutics.^11^, ^12^ However, we found that CLL‐1 expression was lower in the LSC‐enriched CD34^+^CD38^−^ compartment and inversely correlated with the LSC gene signature in CD34‐positive AML samples. Our data therefore corroborate recent findings by Perna et al and Haubner et al, who reported lower percentages of CLL‐1^+^ cells among CD34^+^38^−^ LSC as compared to bulk AML cells^32^, ^33^ as well as findings that low CLL‐1 expression both at the protein and gene expression level was associated with increased LSC frequency.^16^ Altogether, these results support the notion that high CLL‐1 expression is not a suitable marker for identification of leukemic cells with LSC activity among AML bulk cells. These findings may also explain the fact, why CLL‐1‐targeting CAR T cells only displayed modest activity against primary human AML blasts xenografted into immunocompromised mice,^19^ although a CLL‐1 targeting approach has been proven to be very effective in AML cell line xenografts.^13^, ^14^ Interestingly, primary human AML cell killing could be enhanced by CAR T cells targeting a combination of CLL‐1 with other surface markers such as CCR1 or LILRB2.^32^ First feasibility results of an ongoing clinical trial with a combinatorial approach using CAR T cells targeting CLL‐1 as well as CD33 were recently published.^34^

In conclusion, we found that surface expression of GPR56 was high in LSC‐enriched CD34^+^38^−^ leukemic cells and correlated with a LSC gene signature in CD34‐positive AML as well as an adverse clinical outcome. Our data therefore further strengthen the use of GPR56 not only as a marker for LSC activity among bulk leukemia cells in CD34‐positive AML at diagnosis but also as a promising prognostic marker. In contrast, CLL‐1 expression correlated inversely with a LSC gene signature and may therefore have limited potential for identification of LSCs among AML cells. However, due to its aberrant expression on AML cells as compared to normal HSPCs, it may still represent a powerful antigen for combinatorial targeted therapy approaches and may prove useful for residual disease detection by flow cytometry.

## CONFLICT OF INTEREST

BP and AW report research support from Becton Dickinson BioSciences.
