# Supplementary material for: High GPR56 surface expression correlates with a leukemic stem cell gene signature in CD34‐positive AML
Source: Cancer Med. 2019 Mar 7;8(4):1771–8. doi: 10.1002/cam4.2053 (PMC6488118; doi:10.1002/cam4.2053)
Supplement: Supplementary file 1 [file CAM4-8-1771-s001.pdf]

Supporting Information

High GPR56 surface expression correlates with a leukemic stem cell gene signature in CD34-positive AML

Shruti Daga, MSc[1,2], Angelika Rosenberger, BSc[1,2], Franz Quehenberger, PhD[3], Nina Krisper, MSc[2], Barbara Prietl, PhD[2,4], Andreas Reinisch, MD, PhD[1], Armin Zebisch, MD[1], Heinz Sill, MD[1] and Albert Wölfler, MD[1,2]

[1]Division of Hematology, Medical University of Graz, Auenbruggerplatz 38, 8036 Graz, Austria;

[2]CBmed Center of Biomarker Research in Medicine, Stifitingtalstraße 5, 8036 Graz, Austria;

[3]Institute of Medical Informatics, Statistics and Documentation, Medical University of Graz, Auenbruggerplatz 2, 8036 Graz, Austria

[4]Division of Endocrinology and Diabetology, Medical University of Graz, Auenbruggerplatz 15, 8036 Graz, Austria;

Corresponding author:
Albert Wölfler, MD
Division of Hematology, Medical University of Graz,
Auenbruggerplatz 38,
8036 Graz, Austria
Tel: +43 316 385 84084
Fax: +43 316 385 14087
Email: albert.woelfler@medunigraz.at

Figure S1:

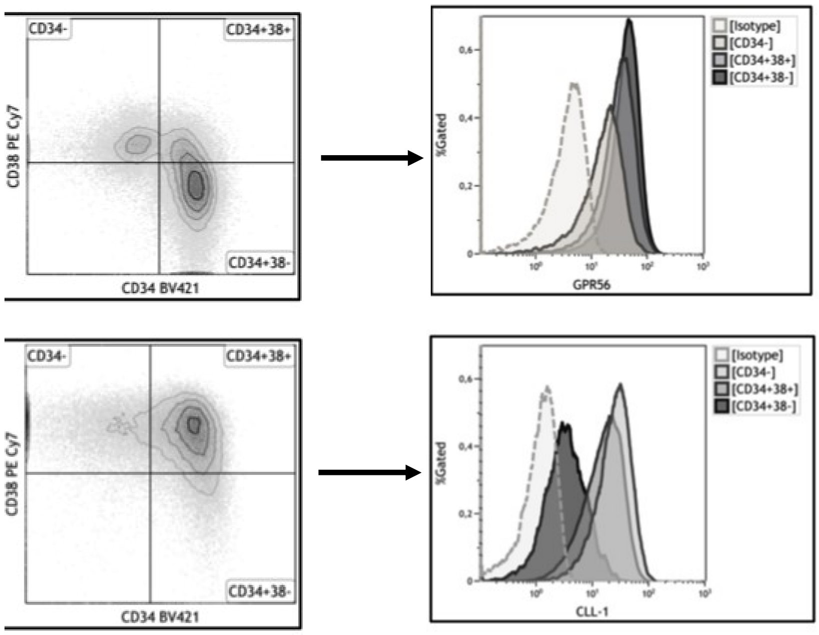

Gating strategy of CD34/38 AML compartments and representative AML samples displaying differential expression of GPR56 and CLL-1, respectively.

Figure S2:

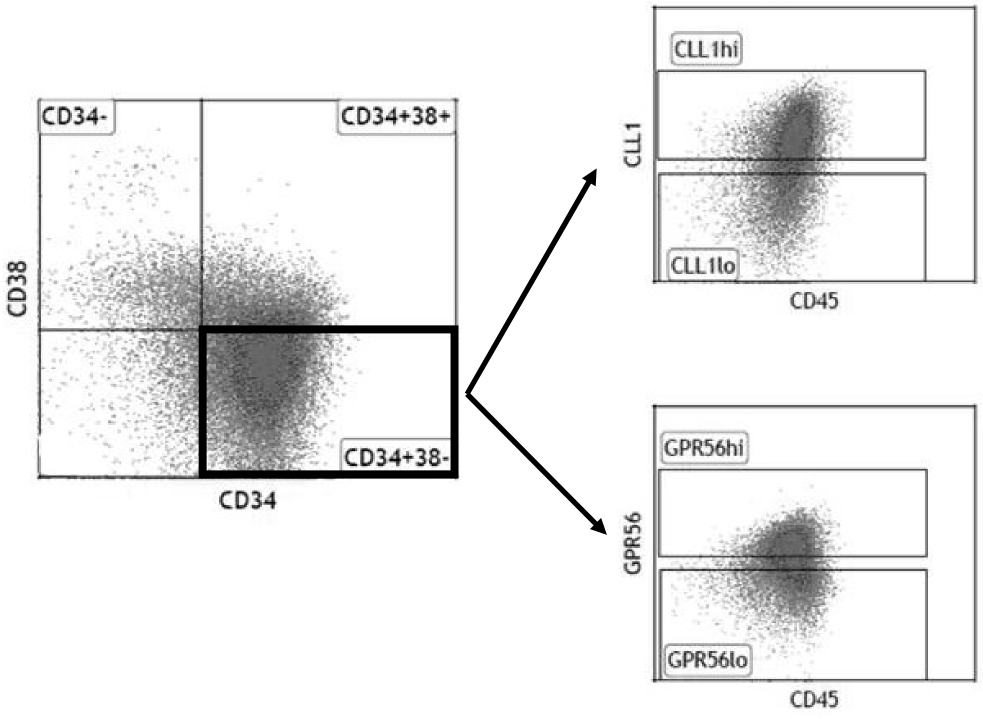

Gating strategy of GPR56^{hi} versus GPR56^{lo} and CLL-1^{hi} versus CLL-1^{lo} CD34^{+}38^{-} AML cells, respectively.

Figure S3:

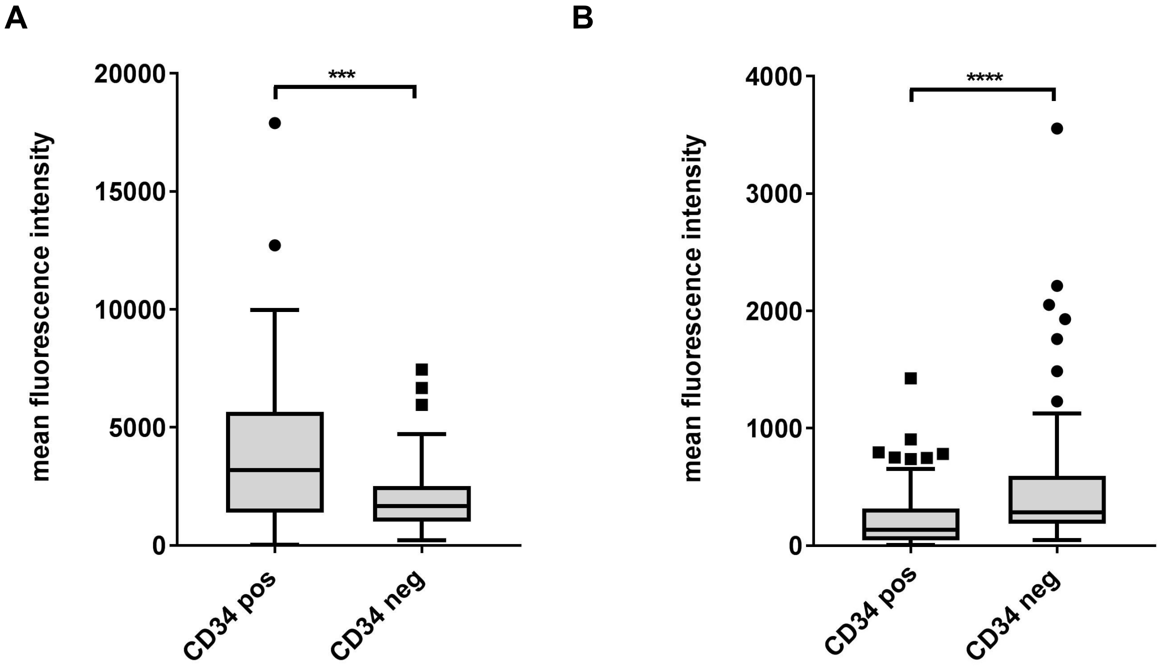

A) Mean fluorescence intensity (MFI) of GPR56 surface expression in CD34-positive (n=108) vs. CD34-negative (n=42) AML samples at diagnosis. GPR56 MFI levels were highly significantly different between both AML groups with higher MFI levels in CD34-positive samples as assessed by the Mann-Withney test (*** p<0.001). **B**) MFI of CLL-1 expression in CD34-positive vs. CD34-negative AML samples at diagnosis. CLL-1 expression was highly significantly different between groups with higher MFI levels in CD34-negative samples as assessed by the Mann-Withney test (**** p<0.0001).

Figure S4:

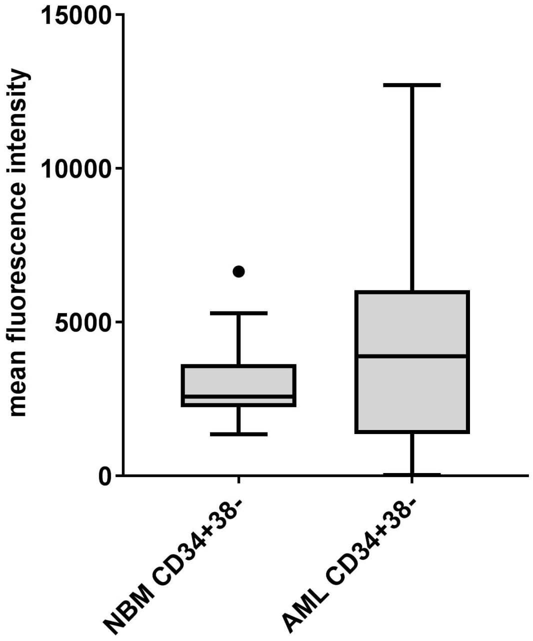

Mean fluorescence intensity (MFI) of GPR56 surface expression in $CD34^{+}38^{-}$ normal bone marrow (NBM) cells (n=16) did not differ from MFI values of $CD34^{+}38^{-}$ AML blasts of CD34-positive AML samples (n=57); p=0.42.

Figure S5:

A **B**

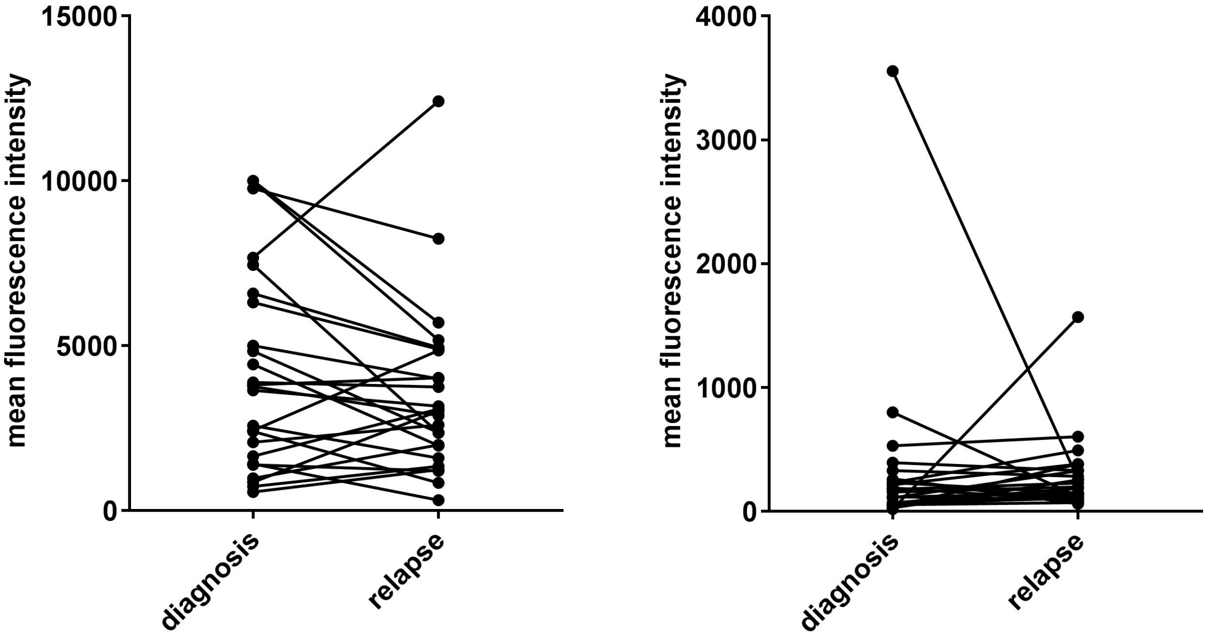

A) Mean fluorescence intensity (MFI) of GPR56 surface expression in 25 diagnostic and relapse samples. GPR56 MFI levels were not significantly different (p=0.25) with stable or increased MFI levels in 21 out of 25 samples. Significant up- or down-regulation was defined by a >2-fold or <0.5-fold change in MFI values, respectively. **B**) MFI of CLL-1 expression in diagnostic and relapse samples. CLL-1 MFI levels were not significantly different (p=0.29) with stable or increased MFI levels in 20/25 samples.

Figure S6:

A

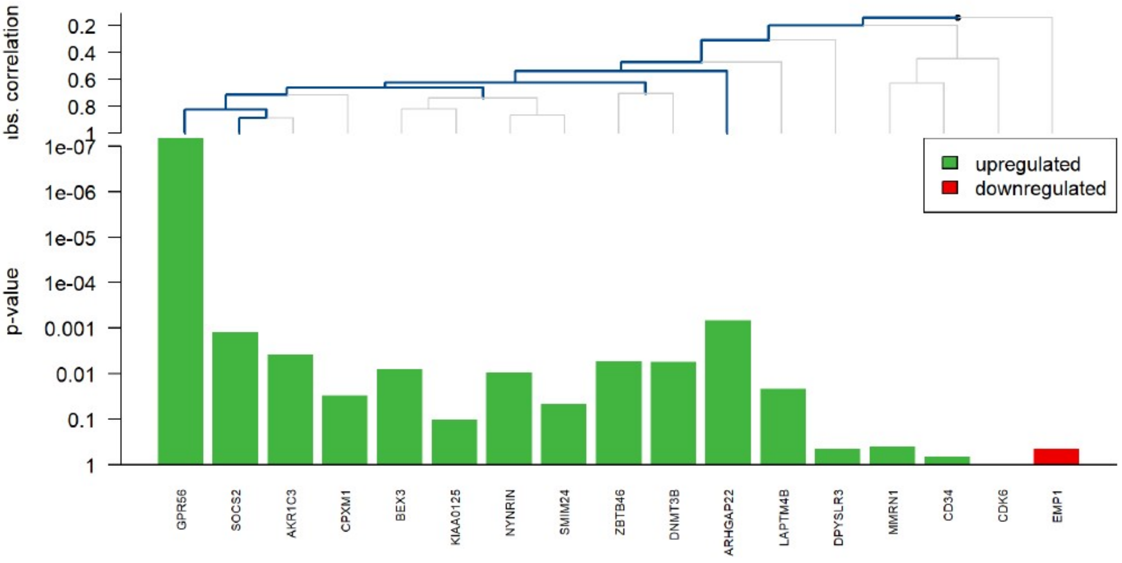

B

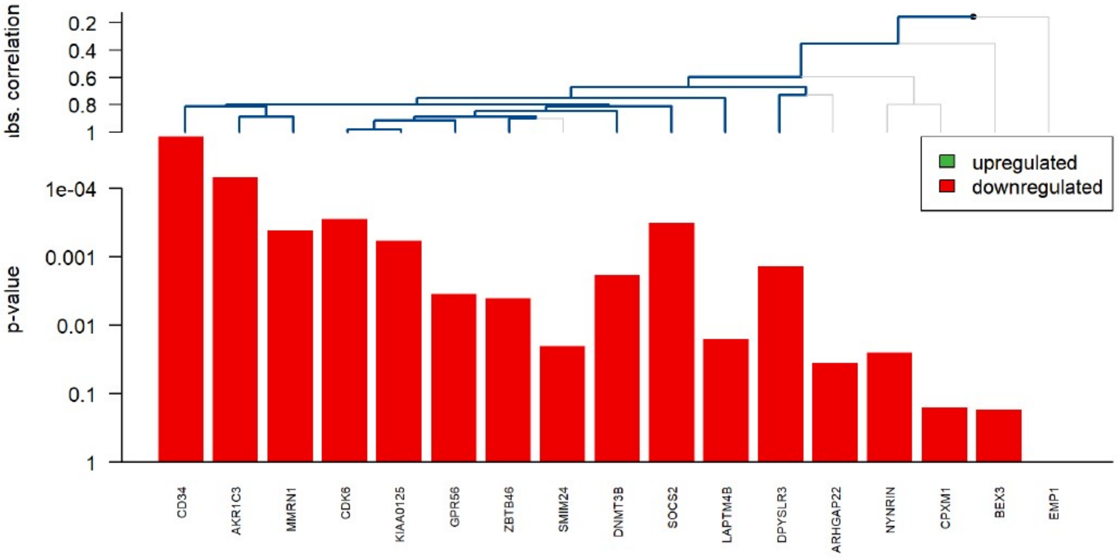

A global test described by Goelman et al. (2007, see reference 23 in main manuscript text), which can assess the association of a group of genes with a distinct phenotype or parameter, showed a highly significant association of high GPR56 surface levels in $CD34^{+}38^{-}$ AML cells with high expression of genes included in the LSC17 panel ($p<0.0001$), even when the *GPR56* gene was excluded ($p<0.001$). This test also allows visualization of the association of single genes with the parameter as well as their similarity in a dendrogram (**A**). Bold lines indicate that the global test for association of the set of genes defined by cluster analysis with GPR56 is significant even after correction for multiple testing.

(**B)** In contrast, high CLL-1 surface levels in $CD34^{+}38^{-}$ AML cells were associated with downregulation of genes included in the LSC17 panel ($p<0.001$).

Table S1: Patient characteristics

	(n=150)
**Gender** (female/male)	66/84
**Age** (median, range in years)	65 (20-93)
age <60 years	65
age ≥60 years	85
**Type of AML**	
de novo AML	88
secondary AML	41
therapy-related AML	21
**WBC** at diagnosis (G/l)	40.8 (0.8-335.4)
WBC <30 G/l	62
WBC ≥30 G/l	86
not known	2
**Cytogenetic risk:**	
favorable risk	10
intermediate risk	76
adverse risk	36
not known	28

Table S2: Antibody panels for multiparameter flow cytometry

Tube 1	CD Marker	Clone	Fluorophore	Company
	CD34	581	BV421	Becton Dickinson (BD)
	CD45RA	HI100	BV510	BD
	CD96	6F9	BV711	BD
	CD47	B6H12	BV786	BD
	CLL-1	50C1	FITC	BD
	7AAD	-	PerCP	BD
	CD366 (TIM3)	7D3	PE	BD
	CD123	7G3	PE-CF594	BD
	CD38	HB7	PE-Cy7	BD
	IL1RAcP	89412	APC	R&D Systems
	CD25	2A3	APC-R700	BD
	CD45	2D1	APC-H7	BD

Tube 2	CD Marker	Clone	Fluorophore	Company
	CD34	581	BV421	BD
	CD33	WM53	BV510	BD
	CD117	104D2	BV650	BD
	CD99	TU12	FITC	BD
	CD44	G44-26	PerCP-Cy5.5	BD
	CD180	G28-8	PE	BD
	CD49f	GOH3	PE-CF594	BD
	CD38	HB7	PE-Cy7	BD
	CD305	DX26	Alexa 647	BD
	CD56	NCAM16.2	APC-R700	BD
	CD45	2D1	APC-H7	BD

Tube 3	CD Marker	Clone	Fluorophore	Company
	CD34	581	BV421	BD
	CD11b	ICRF-44	BV510	BD
	CD14	MφP9	BV711	BD
	CD10	HI10A	BV786	BD
	CD4	SFCI12T4D11	FITC	Beckman Coulter
	GPR56	4C3	PE	Biolegend
	CD19	HIB19	PE-CF594	BD
	CD38	HB7	PE-Cy7	BD
	Jam C	SHM33	APC	Biolegend
	CD7	8H8.1	Alexa 700	Beckman Coulter
	CD45	2D1	APC-H7	BD

	CD Marker	Clone	Fluorophore	Company
**Sorting Tube1**	CD34	581	APC	BD
	CD38	HB7	PE-Cy7	BD
	CD45	2D1	APC-H7	BD
	CLL-1	50C1	PE	BD
	CD14	MφP9	BV421	BD
**Sorting Tube 2**	CD34	581	APC	BD
	CD38	HB7	PE-Cy7	BD
	CD45	2D1	APC-H7	BD
	GPR56	4C3	PE	Biolegend
	CD14	MφP9	BV421	BD

Table S3: Mean fluorescence intensity (MFI) ± standard deviation of other surface markers tested in CD34-positive versus CD34-negative AML samples. Differences in MFI values between groups were analyzed using Mann-Withney test.

marker	CD34-positive AMLs	CD34-negative AMLs	p-value
**CD25**	119.6 ±269.16	120.37 ±260.95	0.5932
**CD33**	783.74 ±835.38	1496.0 ±1179.49	0.0001***
**CD44**	15256.47 ±7552.18	22588.91 ±14617.05	0.0146*
**CD45RA**	2607.0 ±2108.41	1709.69 ±1845.44	0.0097**
**CD47**	266.48 ±271.87	382.22 ±357.51	0.0330*
**CD49f**	884.53 ±921.34	502.10 ±559.77	0.0004***
**CD96**	160.93 ±113.21	152.20 ±97.70	0.9554
**CD99**	705.30 ±463.58	1011.39 ±672.33	0.0065**
**CD117**	3734.63 ±2723.56	1563.71 ±1183.72	<0.0001****
**CD123**	426.40 ±491.11	617.69 ±735.76	0.0381*
**CD305**	1636.58 ±1207.18	2797.77 ±2929.65	0.0095**
**IL-1RAP**	189.56 ±214.99	294.45 ±286.16	0.0373*
**TIM-3**	895.57 ±796.19	876.31 ±1243.32	0.0822
**JAM-C**	747.33 ±610.81	998.69 ±1151.25	0.7356

Table S4: Mean fluorescence intensity (MFI) ± standard deviation of other surface markers tested in CD34/38 compartments of CD34-positive AML samples (n=57). Differences in MFI values between all groups were analyzed using the Kruskal-Wallis test.

marker	$CD34^{+}38^{-}$	$CD34^{+}38^{+}$	$CD34^{-}38^{+}$	p-value
**CD25**	131.89 ±307.88	92.11 ±247.66	160.39 ±326.22	0.0822
**CD33**	752.86 ±662.31	1018.61 ±868.42	1108.49 ±907.74	0.0976
**CD44**	16849.36 ±8007.52	15422.48 ±7859.47	13084.93 ±8370.58	0.0348*
**CD45RA**	2700.16 ±2159.40	2515.54 ±1901.30	2028.51 ±1591.28	0.3047
**CD47**	244.23 ±250.91	340.39 ±392.03	295.46 ±314.29	0.6299
**CD49f**	937.74 ±1100.59	920.14 ±941.50	797.67 ±982.35	0.2528
**CD96**	189.91 ±167.94	200.48 ±277.40	150.52 ±129.29	0.5196
**CD99**	631.16 ±334.92	638.25 ±330.98	515.18 ±314.88	0.0099**
**CD117**	3441.95 ±1985.58	4102.56 ±3469.46	3460.68 ±6635.33	0.0034**
**CD123**	423.30 ±493.60	552.05 ±432.20	662.04 ±709.75	0.0186*
**CD305**	1605.98 ±1165.26	1782.81 ±1364.35	1764.11 ±1455.42	0.9039
**IL-1RAP**	163.68 ±158.28	192.46 ±208.20	177.11 ±179.76	0.9390
**TIM-3**	878.05 ±724.80	966.37 ±738.04	712.0 ±655.80	0.0877
**JAM-C**	742.19 ±582.42	860.40 ±681.89	857.65 ±748.08	0.3301

Table S5: Multivariate analysis of factors affecting overall survival in AML patients having received intensive chemotherapy. GPR56 and CLL-1 expression levels were used as a dichotomous variable using the median value of the cohort as the cut-off level for high versus low expression.

	**hazard ratio (95%CI)**	**p-value**
**WBC**<br>(high vs. low)	1.75 (1.0 - 3.215)	0.064
**Type of AML**<br>(de novo vs. others)	0.57 (0.31 - 1.0)	0.066
**Cytogenetic risk**<br>(high vs. intermediate/good)	1.75 (1.01 - 3.03)	0.044*
**allogeneic SCT**<br>(yes vs. no)	0.48 (0.27 - 0.83)	0.01*
**GPR56**<br>(high vs. low)	1.49 (0.83 - 2.63)	0.17
**CLL-1**<br>(high vs. low)	0.91 (0.5 - 1.56)	0.69
